# Retinal Neurovascular Impairment in Non-diabetic and Non-dialytic Chronic Kidney Disease Patients

**DOI:** 10.3389/fnins.2021.703898

**Published:** 2021-11-18

**Authors:** Xiaomin Zeng, Yijun Hu, Yuanhan Chen, Zhanjie Lin, Yingying Liang, Baoyi Liu, Pingting Zhong, Yu Xiao, Cong Li, Guanrong Wu, Huiqian Kong, Zijing Du, Yun Ren, Ying Fang, Zhiming Ye, Xiaohong Yang, Honghua Yu

**Affiliations:** ^1^Guangdong Eye Institute, Department of Ophthalmology, Guangdong Provincial People’s Hospital, Guangdong Academy of Medical Sciences, Guangzhou, China; ^2^The Second School of Clinical Medicine, Southern Medical University, Guangzhou, China; ^3^Aier Institute of Refractive Surgery, Refractive Surgery Center, Guangzhou Aier Eye Hospital, Guangzhou, China; ^4^Aier School of Ophthalmology, Central South University, Changsha, China; ^5^Division of Nephrology, Guangdong Provincial People’s Hospital, Guangdong Academy of Medical Sciences, Guangzhou, China; ^6^Shantou University Medical College, Shantou, China; ^7^School of Medicine, South China University of Technology, Guangzhou, China

**Keywords:** retinal imaging, neuronal impairment, microvascular hypoperfusion, optical coherence tomography angiography (OCTA), chronic kidney disease

## Abstract

**Background:** Widespread neural and microvascular injuries are common in chronic kidney disease (CKD), increasing risks of neurovascular complications and mortality. Early detection of such changes helps assess the risks of neurovascular complications for CKD patients. As an extension of central nervous system, the retina provides a characteristic window to observe neurovascular alterations in CKD. This study aimed to determine the presence of retinal neurovascular impairment in different stages of CKD.

**Methods:** One hundred fifteen non-diabetic and non-dialytic CKD patients of all stages and a control group of 35 healthy subjects were included. Retinal neural and microvascular parameters were obtained by optical coherence tomography angiography (OCTA) examination.

**Results:** CKD 1–2 group (versus control group) had greater odds of having decreased retinal ganglion cell-inner plexiform layer thickness (GC-IPLt) (odds ratio [OR]: 0.92; 95% confidence interval [CI]: 0.86–0.98), increased ganglion cell complex-focal loss volume (GCC-FLV) (OR: 3.51; 95% CI: 1.27–9.67), and GCC-global loss volume (GCC-GLV) (OR: 2.48; 95% CI: 1.27–4.82). The presence of advanced stages of CKD (CKD 3–5 group versus CKD 1–2 group) had greater odds of having decreased retinal vessel density in superficial vascular plexus (SVP)-WholeImage (OR: 0.77, 95% CI: 0.63–0.92), SVP-ParaFovea (OR: 0.83, 95% CI: 0.71–0.97), SVP-ParaFovea (OR: 0.76, 95% CI: 0.63–0.91), deep vascular plexus (DVP)-WholeImage (OR: 0.89, 95% CI: 0.81–0.98), DVP-ParaFovea (OR: 0.88, 95% CI: 0.78–0.99), and DVP-PeriFovea (OR: 0.90, 95% CI: 0.83–0.98). Besides, stepwise multivariate linear regression among CKD patients showed that β2-microglobulin was negatively associated with GC-IPLt (β: –0.294; 95% CI: –0.469 ∼ –0.118), and parathyroid hormone was positively associated with increased GCC-FLV (β: 0.004; 95% CI: 0.002∼0.006) and GCC-GLV (β: 0.007; 95% CI: 0.004∼0.01). Urine protein to creatinine ratio was positively associated with increased GCC-FLV (β: 0.003; 95% CI: 0.001∼0.004) and GCC-GLV (β: 0.003; 95% CI: 0.001∼0.006).

**Conclusion:** Retinal neuronal impairment is present in early stages of CKD (stages 1–2), and it is associated with accumulation of uremic toxins and higher UACR, while retinal microvascular hypoperfusion, which is associated with worse eGFR, was only observed in relatively advanced stages of CKD (stages 3–5). The results highlight the importance of monitoring retinal neurovascular impairment in different stages of CKD.

## Introduction

As an emerging public health issue, chronic kidney disease (CKD) is predicted to be the fifth most common cause of death worldwide by 2040 (Foreman et al., 2018). Uremia exposure, endocrine failure, and impaired vascular homeostasis result in widespread neural and microvascular injury (Futrakul et al., 2008). In general, neurovascular complications, especially those of the central nervous system (CNS), such as cognitive deterioration (Kurella et al., 2005), cerebrovascular stroke (Smogorzewski, 2001), and encephalopathy (Raskin and Fishman, 1976), are commonly and largely contribute to morbidity and mortality in CKD patients (Wanner et al., 2016). Therefore, there has been an urgent need for early identifying neurovascular impairment in CKD patients (Houben et al., 2017).

As an extension of the brain and sharing the same embryological origin with the CNS. Chua et al. (2020), Kashani et al. (2021), the retina is widely regarded as an accessible source for studying neurodegenerative and vascular injury processes occurring in the CNS (Hart et al., 2016; Mutlu et al., 2018). Consistently, there has also been intense interest in using retinal imaging technology to understand, diagnose, and monitor neurological diseases (Kashani et al., 2021). Recent advancements in optical coherence tomography angiography (OCTA) technologies have allowed for non-invasive and quantitative assessment of the neurovascular structure on different retinal layers (Pujari et al., 2020; Hormel et al., 2021). In some studies, OCTA has been used to monitor retinal neural and microvascular alterations in degenerative neural diseases such as Alzheimer’s disease (O’bryhim et al., 2018; Den Haan et al., 2019) and Parkinson’s disease (Kashani et al., 2021; Robbins et al., 2021).

Previous investigations have shown that both retinal neural impairment and microvascular hypoperfusion can be detected in CKD patients (stages 3–5) using OCTA (Vadala et al., 2019; Yeung et al., 2019; Zhuang et al., 2020). However, those studies failed to included CKD patients at the early stages (stages 1–2), so it still reminds unknown whether retinal neurovascular impairment occurs in earlier stages of CKD and what are the related factors for such damage. Furthermore, the inclusion of diabetes mellitus (DM) patients and dialysis population in prior studies challenge the direct comparison between CKD patients and normal subjects (Yeung et al., 2019; Wu et al., 2020), as both DM (Zeng et al., 2019; Zhuang et al., 2020) and dialysis (Wu et al., 2020) can deteriorate neurovascular system independent of declining renal function.

Therefore, we conducted this cross-sectional study in CKD patients of all stages without a history of DM or dialysis, aiming to investigate the changes of retinal neurovascular parameters detected by OCTA in patients of different CKD stages and further analyze the association between neurovascular alterations and CKD-related risk factors.

## Materials and Methods

### Design and Population of the Study

This cross-sectional study included a total of 150 eyes from 115 CKD patients and 35 healthy subjects. The study was conducted in the Department of Ophthalmology and the Department of Nephrology, Guangdong Provincial People’s Hospital from August 2019 to December 2020. The procedures followed the ethical standards of the Research Ethics Committee of Guangdong Provincial People’s Hospital [registration number: GDREC2020069(R1)] and the Helsinki Declaration. This study followed the Strengthening the Reporting of Observational Studies in Epidemiology (STROBE) reporting guideline. Informed consent was obtained from all participants.

The inclusion criteria were patients with CKD and aged ≥ 18 years. The definition of CKD was based on the presence of kidney damage (i.e., albuminuria or urinary albumin to creatinine ratio) or decreased kidney function [i.e., estimated glomerular filtration rate (eGFR) < 60 ml/min/1.73 m^2^ for 3 months or more] (Levey and Coresh, 2012). The eGFR value was calculated from serum creatinine (Scr) using the CKD-EPI creatinine equation (Levey et al., 2009). Severity of CKD was categorized based on the eGFR values: more than 90 ml/min/1.73 m^2^ (stage 1), 60∼89 ml/min/1.73 m^2^ (stage 2), 30∼59 ml/min/1.73 m^2^ (stage 3), 15∼29 ml/min/1.73 m^2^ (stage 4), and less than 15 ml/min/1.73 m^2^ (stage 5) (Levey and Coresh, 2012). The CKD 1–2 group were CKD patients in stages 1–2, and the CKD 3–5 group were CKD patients in stages 3–5. The control group was healthy subjects without major systemic diseases or ocular diseases.

The exclusion criteria were patients with (1) any type of DM; (2) HblAc>6.5%; (3) history of dialysis (hemodialysis or peritoneal dialysis); (4) any ocular issue that may impair ocular circulation (e.g., glaucoma, eye trauma, retinal vascular occlusion, choroidal neovascularization, endophthalmitis, or refractive error > ± 6 diopters); (5) inadequate quality of OCTA image (quality score < 6 or the presence of significant artifact); (6) any severe systemic diseases (e.g., cerebral infarction, myocardial infarction, heart failure, or connective tissue disorder); (7) women who were pregnant.

### Ophthalmic Examinations

All participants underwent comprehensive ophthalmic examinations including best-corrected visual acuity (BCVA) (measured on a decimal chart and presented as logMAR), autorefraction, intraocular pressure, slit-lamp examination, and color fundus photography. Retinal microvasculature and neural parameters were measured with Optical Coherence Tomography Angiography (OCTA).

Optical Coherence Tomography Angiography with RTVue-XR Avanti (Optovue, Fremont, CA, United States, version 2018) is a device combining structural and functional imaging by analyzing the changing variance in light speckle created by erythrocyte flow over multiple scans (Spaide et al., 2018). The OCTA machine generates a contract-free angiogram down to the capillary level and surrogate indices of perfusion. The OCTA platform has been integrated with split-spectrum amplitude-decorrelation angiography (SSADA) algorithm that automatically segments OCT images alongside angiographic data to report global and regional vessel density (VD) of each retinal layer (Hormel et al., 2021).

The detailed procedures of the OCTA examination were shown in Supplementary Figure S1. The OCTA examination was performed in a darkroom. Before examination, participants’ pupils were dilated with Tropicamide Phenylephrine Eye Drops. An internal fixation light was used as the center the scanning area. The OCT signal position and quality were optimized using the Auto All function, aiming to find the best position, focus, and polarization match for obtaining the retina OCT image (Toto et al., 2016). Besides, considering that media opacity made an effect of on the measurement of retinal neurovascular parameters (Zhang et al., 2020, 2021), the OCTA system generated a signal strength index (SSI) to help determine whether the scan quality is acceptable. The signal strength intensity (SSI; ranged from 0 to 100) is based on the intensity or brightness of the reflected light during scanning. The higher the intensity, the higher the SSI.

Retinal vascular retinal layers were visualized as follows: a set of high definition (HD) Angio Retina Scan (6*6mm) was captured to evaluate the VD of the macular, which contained superficial retinal plexus and deep retinal plexus. The boundaries of each layer were segmented as follows: a slab extending from 3 to 15 μm from the internal limiting membrane (ILM) was generated to detect the superficial vascular plexus (SVP), and a slab extending from 15 to 70 μm below ILM to detect the deep vascular plexus (DVP) (Zhuang et al., 2020). Besides, HD Angio Disk Scan (4.5*4.5 mm) was imaged to visualize the radial peripapillary capillary, which was defined as the vessel on the layer between the outer limit of the retinal nerve fiber layer (RNFL) and ILM in the peripapillary region. The segmentations of these three retinal vascular plexuses were shown in Supplementary Figure S2 (Wu et al., 2020).

As for the retinal neural parameters, a ganglion cell complex (GCC) scan was used to measure retinal ganglion cell-inner plexiform layer thickness (GC-IPLt), which was defined as the layer between the retinal nerve fiber layer (RNFL) and IPL within a 6-mm circle. An optic nerve head (ONH) scan was obtained for the measurement of RNFL thickness (RNFLt). The average RNFLt was measured over a 3.45-mm-diameter circle centered on the ONH.

All the retinal VD and layer thickness retinal VD and layer thickness were calculated automatically and quantitatively using the AngioVue SSADA software (Peng et al., 2020; Wu et al., 2020). Both eyes of the participants were examined by OCTA, but only the data of the right eye was included for analysis. If the scan of the right eye was uninterpretable, data of the left eye were utilized. Only images with quality index ≥ 6 and SSI ≥ 60 were retained.

### Systemic Data Collection

Medical history and laboratory data in the last 3 months were collected. Demographic and blood test data were extracted from the electronic medical record system by a trained researcher (YF) and double-checked by an ophthalmologist (XZ). The basic information included age, sex, body mass index (BMI), systolic blood pressure (SBP), diastolic blood pressure (DBP), duration of CKD, and the history of smoking, hypertension, and cardiovascular disease (CVD). Laboratory tests included glycated hemoglobin (HbA_1_C), Scr, blood urea nitrogen (BUN), hemoglobin (HGB), cholesterol (CHOL), low-density lipoprotein (LDL), urine albumin to creatinine ratio (UACR), urine protein to creatinine ratio (UPCR), parathyroid hormone (PTH), albumin (ALB), and β2-microglobulin (β2-M).

### Statistical Analysis

SPSS version 25.0 (SPSS. Inc., Chicago, IL, United States) was used to perform statistical analyses. A two-sided *p*-value of less than 0.05 was considered significant. Mean and standard deviation (SD) were used for presenting quantitative variables for normal distribution, medians and interquartile range (IQR) for abnormal distribution, and numbers (percentages) were used for categorical variables. In the comparison of basic characteristics among three groups, one-way ANOVA was used for normally distributed continuous data and the Kruskal–Wallis *H* test was used for non-normally distributed continuous data, and Bonferroni’s correction was used for *post hoc* analysis. χ2 tests were utilized to compare categorical variables. Mann–Whitney test was applied to compare the duration of CKD between the CKD 1–2 group and the CKD 3–5 group. Crude logistic regression models were used to compare the OCTA parameters (dependent variables) between different groups (independent variables, 1^*a*^: CKD 1–2 versus control, 2^*a*^: CKD 1–2 versus CKD 3–5) without adjusting confounders. We also used adjusted logistic regression models (1^*b*^: CKD 1–2 versus control, 2^*b*^: CKD 3–5 versus CKD 1–2) to adjust the confounders, including age, sex, BMI, SBP, DBP, history of smoking, history of CVD, Hb1Ac, CHOL, and LDL. Partial correlation analysis was used to determine risk factors associated with the OCTA parameters in the CKD patients after controlling for age, sex, BMI, history of smoking, history of CVD, RE, and IOP. Besides, stepwise multivariate regression was performed to figure out the independent risk factors for the retinal neurovascular alterations in the CKD patients. Age, sex, BMI, history of hypertension, history of smoking, history of CVD, eGFR, BUN, PTH, UACR, HGB, CKD duration, Hb1Ac, β2-M, and LDL were independent variables entered into the model. Finally, Spearman correlation analysis was used to determine the relationship between BCVA and OCTA parameters in all subjects.

## Results

### Basic Characteristics Among the Three Groups

The basic characteristics of the participants were summarized in Table 1. The etiology of the CKD patients was shown in Supplementary Table S1. There were significantly higher results of DBP, BUN, and HGB in the CKD 1–2 group compared to the control group (all *p* < 0.05). The CKD 3–5 group showed significantly higher results of SBP, DBP, Hb1Ac, Scr, BUN, BCVA (logMAR), as well as decreased eGFR and HGB compared to the CKD 1–2 group and the control group (all *p* < 0.05). Besides, the CKD 3–5 group also showed significantly higher results of age, BMI, PTH, β2-M, history of hypertension, and CVD compared to the CKD 1–2 group (all *p* < 0.05). No significant difference was found in sex, history of smoking, CHOL, SER, and IOP among the three groups. Duration of CKD, UACR, UPCR, and ALB and the etiology of CKD were comparable between the CKD 1–2 group and the CKD 3–5 group. Besides, the signal strength index of the macular, disk, GGG, and ONH areas and the overall quality index of the macular and disk areas are comparable among the three groups (Supplementary Table S2).

**TABLE 1:** Comparison of basic characteristics among the three groups.

	Control (*n* = 35)	CKD 1–2 (*n* = 50)	CKD 3–5 (*n* = 65)	*p*	*p* ^a^	*p* ^b^	*p* ^c^
**Basic characteristics**							
Age, year	45.06 ± 11.28	40.24 ± 13.40	47.58 ± 12.76	**0.010**^δ^	0.259	1.000	**0.007**
Sex, male, n (%)	15 (42.90)	20 (40.00)	38 (58.50)	0.107^†^	–	–	–
BMI, kg/m^2^	22.18 ± 2.11	22.36 ± 3.65	23.88 ± 3.09	**0.009** ^δ^	0.989	0.059	**0.005**
SBP, mmHg	118 ± 11.43	124.32 ± 13.12	141.98 ± 21.3	**<0.001** ^δ^	0.061	**<0.001**	**<0.001**
DBP, mmHg	73 ± 6.06	80.02 ± 11.27	85.88 ± 12.89	**<0.001** ^δ^	**0.001**	**<0.001**	**0.032**
History of smoking, *n* (%)	4 (11.40)	6 (12.00)	11 (16.90)	0.664^†^	–	–	–
History of hypertensive, *n* (%)	–	10 (20.00)	46 (70.80)	**<0.001** ^†^	–	–	–
History of CVD, *n* (%)	–	2 (4.00)	12 (18.50)	**0.019** ^†^	–	–	–
Duration of CKD, month	–	12 (16.25)	12 (27.00)	0.132^‡^	–	–	–
**Laboratory test**							
Scr, μmol/L	70.36 ± 14.61	73.2 ± 22.69	358.44 ± 316.13	**<0.001** ^δ^	0.862	**<0.001**	**<0.001**
eGFR, ml/min/1.73 m^2^	100.7 ± 20.88	99.02 ± 25.25	27.26 ± 17.60	**<0.001** ^δ^	0.976	**<0.001**	**<0.001**
BUN, mmol/L	5.29 (2.15)	5.78 (2.55)	11.82 (11.09)	**<0.001** ^ξ^	**0.005**	**<0.001**	**<0.001**
Hb1Ac, (%)	5.18 ± 0.41	5.36 ± 0.53	5.60 ± 0.48	**<0.001** ^δ^	0.281	**<0.001**	**0.020**
HGB, 10^9/L	137.33 ± 15.10	124.66 ± 19.74	104.94 ± 23.65	**<0.001** ^δ^	**0.004**	**<0.001**	**<0.001**
CHOL, mmol/L	4.89 ± 0.68	5.71 ± 2.60	5.59 ± 1.83	0.133^δ^	0.174	0.270	1.000
LDL, mmol/L	3.10 ± 0.85	3.72 ± 1.75	3.86 ± 1.27	**0.030** ^δ^	0.124	**0.029**	1.000
UACR, mg/g	–	794.20 (1414.13)	1274.06 (1966.94)	0.163^‡^	–	–	–
UPCR, mg/g	–	1150.50 (2499.47)	2249.44 (2856.25)	0.052^‡^	–	–	–
PTH, pg/ml	–	35.38(12.93)	92.78(113.20)	**<0.001^‡^**	–	–	–
ALB, g/L	–	35.21(11.50)	37.02(12.88)	0.197^‡^	–	–	–
β2-M, μg/ml	–	2.30(1.39)	8.85(10.79)	**<0.001^‡^**	–	–	–
**Ocular characteristics**							
BCVA, LogMAR	–0.0146 ± 0.0671	–0.0087 ± 0.0841	0.0283 ± 0.1208	**<0.001** ^δ^	0.997	**<0.001**	**<0.001**
RE, diopters	–0.99 ± 2.18	–1.27 ± 3.58	–0.55 ± 2.28	0.371^δ^	1.000	1.000	0.496
IOP, mmHg	14.15 ± 2.20	14.16 ± 3.83	13.31 ± 3.30	0.291^δ^	1.000	0.655	0.505

*CKD, chronic kidney disease; BMI, body mass index; SBP, systolic blood pressure; DBP, diastolic blood pressure; CVD, cardiovascular disease; Scr, serum creatinine; eGFR, estimated glomerular filtration rate; BUN, blood urea nitrogen; HbA1c, glycated hemoglobulin; HGB, hemoglobin; CHOL, cholesterol; LDL, low-density lipoprotein; UACR, urine albumin to creatinine ratio; UPCR, urine protein to creatinine ratio; PTH, parathyroid hormone; ALB, albumin; β2-M, β2-microglobulin; BCVA, best-corrected visual acuity; RE, refraction error; IOP, intraocular pressure.*

*Results are presented as mean ± standard deviation, medians (IQR), or as numbers (percentages).*

*A, p (δ One-Way ANOVA and ξ Kruskal–Wallis H test) for the comparison among three groups. p (^†^χ^2^ test and ‡ Mann–Whitney Test) for the comparison between the CKD 1∼2 group and the CKD 3∼5 group.*

*B, p^a^ for the comparison between the control group and the CKD 1∼2 group using post hoc analysis.*

*C, p^b^ for the comparison between the control group and the CKD 3∼5 group using post hoc analysis.*

*D, p^c^ for the comparison between the CKD 1∼2 group and the CKD 3∼5 group using post hoc analysis.*

*The bold values indicated statistically significant (p value < 0.05).*

### Comparisons of Optical Coherence Tomography Angiography Parameters Among the Three Groups

**FIGURE 1:**
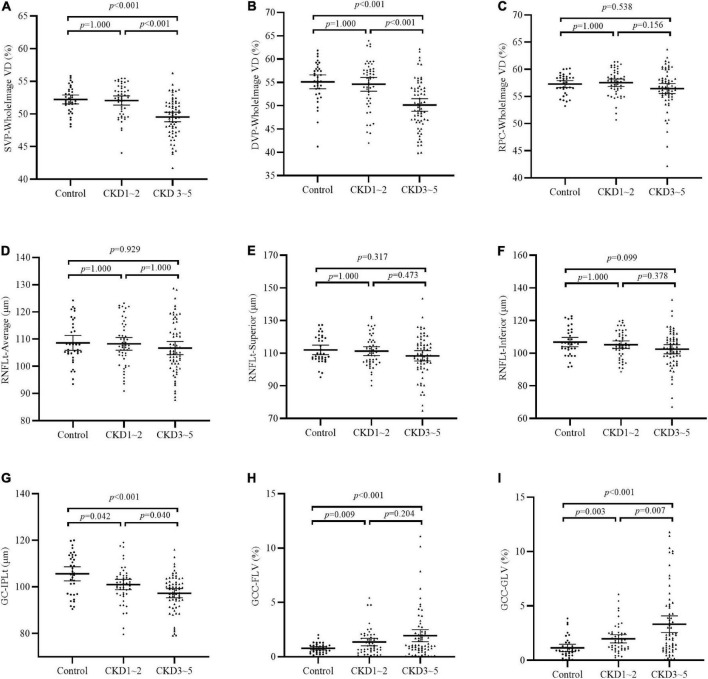
Comparison of OCTA parameters among the three groups. **(A–C)** VD of SVP-WholeImage, DVP-WholeImage, and RPC-WholeImage among three groups; **(D–F)** Thickness of RNFL-Average, RNFL-superior, and RNFL-inferior among three groups; **(G)** Thickness of GC-IPL among three groups; **(H)** Focal loss volume of GCC among three groups; **(I)** Global loss volume of GCC among three groups. CKD, chronic kidney disease; VD, vessel density; SVP, superficial vascular plexus; DVP, deep vascular plexus; RPC, retinal peripapillary capillary; RNFLt, retinal nerve fiber layer thickness; GC-IPLt, ganglion cell-inner plexiform layer thickness; GCC, ganglion cell complex; GLV, global loss volume; FLV, focal loss volume.

Comparisons of OCTA parameters among the three groups were shown in Table 2 and Figure 1. Significantly reduced GC-IPLt and increased GCC-FLV and GCC-GLV were detected in the CKD 1–2 group and the CKD 3–5 group compared to the control group (all *p* < 0.05). Also, significantly reduced GCCt and increased GCC-GLV were found in the CKD 3–5 group compared to the CKD 1–2 group. A representative picture showing the changes in GC-IPLt, GCC-FLV, and GCC-GLV in CKD groups and control group was shown in Supplementary Figure S3. No significant difference was found in RNFL parameters among the three groups. For retinal microvascular parameters, significantly reduced VD in all SVP regions and DVP regions were found in the CKD 3–5 group compared to the CKD 1–2 group and control group (all *p* < 0.001). No significant difference was found in all microvascular parameters between the CKD 1–2 group and the control group. No significant difference was found in VD in all RPC regions among the three groups.

**TABLE 2:** Comparison of OCTA parameters among the three groups.

	Control (*n* = 35)	CKD 1–2 (*n* = 50)	CKD 3–5 (*n* = 65)	*p*	*p* ^a^	*p* ^b^	*p* ^c^
**Retinal microvascular parameters**							
SVP-WholeImage, %	52.22 ± 1.98	52.06 ± 2.44	49.53 ± 2.92	**<0.001**	1.000	**<0.001**	**<0.001**
SVP-ParaFovea, %	54.90 ± 1.95	54.46 ± 2.81	51.28 ± 4.19	**<0.001**	1.000	**<0.001**	**<0.001**
SVP-PeriFovea, %	53.15 ± 2.00	53.01 ± 2.44	50.43 ± 2.94	**<0.001**	1.000	**<0.001**	**<0.001**
DVP-WholeImage, %	55.12 ± 4.30	54.60 ± 5.15	50.15 ± 5.36	**<0.001**	1.000	**<0.001**	**<0.001**
DVP-ParaFovea, %	57.83 ± 2.98	57.51 ± 4.09	53.35 ± 4.76	**<0.001**	1.000	**<0.001**	**<0.001**
DVP-PeriFovea, %	56.77 ± 4.62	56.28 ± 5.69	51.13 ± 6.03	**<0.001**	1.000	**<0.001**	**<0.001**
RPC-WholeImage, %	57.28 ± 1.85	57.53 ± 2.40	56.44 ± 3.70	0.124	1.000	0.538	0.156
RPC-WholeImage-Capillary, %	50.28 ± 1.85	50.64 ± 2.36	50.19 ± 3.50	0.686	1.000	1.000	1.000
**Retinal neural parameters**							
RNFLt-Average, μm	108.63 ± 8.04	108.28 ± 8.26	106.74 ± 9.65	0.506	1.000	0.929	1.000
RNFLt-Superior, μm	112.06 ± 8.45	111.25 ± 9.62	108.36 ± 12.7	0.188	1.000	0.317	0.473
RNFLt-Inferior, μm	106.80 ± 8.35	105.24 ± 8.07	102.45 ± 11.25	0.143	1.000	0.099	0.378
GC-IPLt, μm	105.60 ± 8.77	100.98 ± 7.79	97.27 ± 7.89	**0.004**	**0.042**	**<0.001**	**0.040**
GCC-FLV, %	0.78 ± 0.44	1.34 ± 1.19	1.94 ± 2.26	**<0.001**	**0.009**	**<0.001**	0.204
GCC-GLV, %	1.14 ± 0.98	1.98 ± 1.29	3.32 ± 3.09	**<0.001**	**0.003**	**<0.001**	**0.007**

*OCTA, optical coherence tomography angiography; CKD, chronic kidney disease; SVP, superficial vascular plexus; DVP, deep vascular plexus; RPC, retinal peripapillary capillary; RNFLt, retinal nerve fiber layer thickness; GC-IPLt, ganglion cell-inner plexiform layer thickness; GCC, ganglion cell complex; GLV, global loss volume; FLV, focal loss volume.*

*A, *p* for the comparison among the three groups using One-Way ANOVA.*

*B, *p*^*a*^ for the comparison between the control group and the CKD 1∼2 group using *post hoc* analysis.*

*C, *p*^*b*^ for the comparison between the control group and the CKD 3∼5 group using *post hoc* analysis.*

*D, *p*^*c*^ for the comparison between the CKD 1∼2 group and the CKD 3∼5 group using *post hoc* analysis.*

*The bold values indicated statistically significant (p value < 0.05).*

### Independent Associations Between Optical Coherence Tomography Angiography Parameters and Severity of Chronic Kidney Disease

Table 3 shows the results of logistic regression models investigating associations of OCTA parameters and the incidence and progression of CKD. After adjusting for confounders including age, sex, BMI, SBP, DBP, history of smoking, history of CVD, Hb1Ac, CHOL, and LDL, the model 1^*b*^ showed that the presence of early stages of CKD (CKD1–2 group versus control group) was significantly associated with decreased GC-IPLt (odds ratio [OR]: 0.92; 95% confidence interval [CI]: 0.86–0.98), meaning one-micrometer increase of GC-IPLt is associated with 0.92-fold risk of CKD after adjusting for the confounding factors. Meanwhile, the presence of early stages of CKD (CKD1–2 group versus control group) was significantly associated with increased GCC-FLV (OR: 3.51; 95% CI: 1.27–9.67) and GCC-GLV (OR: 2.48; 95% CI: 1.27–4.82). Further investigation in the crude model 2^*b*^ showed that the presence of advanced stages of CKD (CKD 3–5 group versus CKD 1–2 group) had greater odds of having decreased VD in SVP-WholeImage (OR: 0.77; 95% CI: 0.63–0.92), SVP-ParaFovea (OR: 0.83; 95% CI: 0.71–0.97), SVP-PeriFovea (OR: 0.76; 95% CI: 0.63–0.91), DVP-WholeImage (OR: 0.89; 95% CI: 0.81–0.98), DVP-ParaFovea (OR: 0.88; 95% CI: 0.78–0.99), DVP-PeriFovea (OR: 0.90; 95% CI: 0.83–0.98), and increased GCC-GLV (OR: 1.35; 95% CI: 1.05–1.73).

**TABLE 3:** Association between OCTA parameters and severity of CKD.

	Model 1 (CKD 1–2 group vs. control group)				Model 2 (CKD 1–2 group vs. CKD 3–5 group)			
	Crude model 1^a^		Model 1^b^		Crude model 2^a^		Model 2^b^	
	OR (95% CI)	*p*	OR (95% CI)	*p*	OR (95% CI)	*p*	OR (95% CI)	*p*
SVP-WholeImage	0.97 (0.80–1.18)	0.742	0.97 (0.77–1.23)	0.788	0.70 (0.59–0.83)	**<0.001**	0.77 (0.63–0.92)	**0.005**
SVP-ParaFovea	0.93 (0.77–1.11)	0.418	0.93 (0.73–1.17)	0.526	0.75 (0.65–0.87)	**<0.001**	0.83 (0.71–0.97)	**0.022**
SVP-PeriFovea	0.97 (0.80–1.18)	0.777	0.94 (0.75–1.19)	0.632	0.70 (0.59–0.83)	**<0.001**	0.76 (0.63–0.91)	**0.003**
DVP-WholeImage	0.98 (0.89–1.07)	0.621	0.98 (0.87–1.11)	0.758	0.85 (0.73–0.89)	**<0.001**	0.89 (0.81–0.98)	**0.019**
DVP-ParaFovea	0.98 (0.87–1.10)	0.688	0.98 (0.82–1.18)	0.865	0.81 (0.77–0.93)	**<0.001**	0.88 (0.78–0.99)	**0.037**
DVP-PeriFovea	0.98 (0.90–1.07)	0.669	0.99 (0.89–1.11)	0.856	0.86 (0.80–0.93)	**<0.001**	0.90 (0.83–0.98)	**0.017**
RPC-WholeImage	1.05 (0.86–1.29)	0.599	1.01 (0.79–1.31)	0.908	0.89 (0.78–1.01)	0.080	0.95 (0.81–1.12)	0.519
RPC-WholeImage-Capillary	1.08 (0.88–1.33)	0.439	1.02 (0.78–1.33)	0.876	0.95 (0.84–1.08)	0.435	0.97 (0.82–1.15)	0.736
RNFLt-Average	0.99 (0.94–1.05)	0.845	0.95 (0.89–1.02)	0.189	0.98 (0.94–1.02)	0.366	0.98 (0.93–1.03)	0.432
RNFLt-Superior	0.99 (0.94–1.04)	0.687	0.96 (0.90–1.02)	0.186	0.98 (0.95–1.01)	0.183	0.97 (0.93–1.01)	0.172
RNFLt-Inferior	0.98 (0.93–1.03)	0.385	0.94 (0.88–1.02)	0.122	0.98 (0.94–1.01)	0.223	0.98 (0.94–1.02)	0.360
GC-IPLt	0.92 (0.88–0.99)	**0.016**	0.92 (0.86–0.98)	**0.015**	0.94 (0.89–0.99)	**0.017**	0.94 (0.88–1.00)	0.061
GCC-FLV	2.43 (1.19–4.97)	**0.015**	3.51 (1.27–9.67)	**0.015**	1.22 (0.96–1.54)	0.109	1.21 (0.91–1.60)	0.185
GCC-GLV	2.07 (1.26–3.42)	**0.004**	2.48 (1.27–4.82)	**0.007**	1.30 (1.07–1.58)	**0.009**	1.35 (1.05–1.73)	**0.018**

*OCTA, optical coherence tomography angiography; CKD, chronic kidney disease; OR, odds ratio; CI, confidence interval; SVP, superficial vascular plexus; DVP, deep vascular plexus; RPC, retinal peripapillary capillary; RNFLt, retinal nerve fiber layer thickness; GC-IPLt, ganglion cell-inner plexiform layer thickness; GCC, ganglion cell complex; GLV, global loss volume; FLV, focal loss volume.*

*Model 1: Binary logistics regression model with enter method in the control group and the CKD 1∼2 group.*

*Model 2: Binary logistics regression model with enter method in the CKD 1∼2 group and the CKD 3∼5 group.*

*Model 1^*b*^ and Model 2^*b*^ were adjusted for age, sex, body mass index, systolic blood pressure, diastolic blood pressure, history of smoking, history of cardiovascular disease, glycated hemoglobulin, cholesterol, and low-density lipoprotein.*

*The bold values indicated statistically significant (p value < 0.05).*

### Partial Correlation Between Optical Coherence Tomography Angiography Parameters and Chronic Kidney Disease-Related Parameters

The results of partial correlation analyses of OCTA parameters and CKD-related parameters among all 115 CKD patients were shown in Table 4. The results showed that all retinal microvascular parameters as well as GC-IPLt were positively correlated with eGFR and HGB (*r* = 0.199∼0.355, all *p* < 0.05) and negatively with β2-M, BUN, and PTH (*r* = –0.185 ∼ –0.354, all *p* < 0.05). Furthermore, GCC-FLV and GCC-GLV were positively correlated with β2-M, BUN, UPCR, UACR, and PTH (*r* = 0.193∼0.344, all *p* < 0.05) and negatively with eGFR (*r* = −0.200∼−0.276, all *p* < 0.05). Partial correlation of GCC variables and eGFR were shown in Supplementary Figure S4.

**TABLE 4:** Partial correlation between OCTA parameters and CKD-related data (115 CKD patients).

	eGFR	β2-M	BUN	UPCR	UACR	PTH	HGB
SVP-WholeImage	**(0.283, 0.003)**	**(–0.222, 0.022)**	**(–0.325, 0.001)**	(–0.066, 0.504)	(–0.058, 0.557)	**(–0.349, <0.001)**	**(0.212, 0.029)**
SVP-ParaFovea	**(0.322, 0.001)**	**(–0.188, 0.046)**	**(–0.354, <0.001)**	(–0.014, 0.885)	(0.008, 0.935)	**(–0.271, 0.005)**	**(0.206, 0.034)**
SVP-PeriFovea	**(0.314, 0.001)**	**(–0.196, 0.044)**	**(–0.348, <0.001)**	(–0.055, 0.577)	(–0.035, 0.719)	**(–0.252, 0.009)**	**(0.199, 0.04)**
DVP-WholeImage	**(0.294, 0.002)**	**(–0.222, 0.022)**	**(–0.189, 0.041)**	(–0.002, 0.984)	(0.027, 0.787)	**(–0.185, 0.049)**	**(0.295, 0.002)**
DVP-ParaFovea	**(0.355, <0.001)**	**(–0.275, 0.004)**	**(–0.280, 0.004)**	(–0.025, 0.795)	(0.004, 0.965)	**(–0.267, 0.006)**	**(0.283, 0.003)**
DVP-PeriFovea	**(0.297, 0.002)**	**(–0.233, 0.016)**	**(–0.191, 0.050)**	(–0.005, 0.956)	(0.019, 0.846)	**(–0.200, 0.04)**	**(0.295, 0.002)**
GCI-IPLt	**(0.222, 0.022)**	**(–0.314, 0.001)**	**(–0.285, 0.003)**	(0.016, 0.869)	(0.037, 0.708)	**(–0.221, 0.023)**	**(0.249, 0.01)**
GCC-FLV	**(–0.200, 0.040)**	**(0.252, 0.009)**	**(0.214, 0.027)**	**(0.264, 0.006)**	**(0.255, 0.008)**	**(0.254, 0.009)**	(–0.119, 0.226)
GCC-GLV	**(–0.276, 0.004)**	**(0.344, < 0.001)**	**(0.262, 0.007)**	**(0.232, 0.017)**	**(0.193, 0.047)**	**(0.319, 0.001)**	(–0.181, 0.063)

*OCTA, optical coherence tomography angiography; CKD, chronic kidney disease; eGFR, estimated glomerular filtration rate,* β*2-M,* β*2-microglobulin; BUN, blood urea nitrogen; UACR, urine albumin to creatinine ratio; UPCR, urine protein to creatinine ratio; PTH, parathyroid hormone; HGB, hemoglobin; SVP, superficial vascular plexus; DVP, deep vascular plexus; GC-IPLt, ganglion cell-inner plexiform layer thickness; GCC, ganglion cell complex; GLV, global loss volume; FLV, focal loss volume.*

*Partial correlation adjusted for age, sex, body mass index, history of smoking, history of cardiovascular disease, refraction error, and intraocular pressure.*

*The bold values indicated statistically significant (p value < 0.05).*

### Stepwise Multivariate Linear Regression Between Optical Coherence Tomography Angiography Parameters and Chronic Kidney Disease-Related Parameters

The result of stepwise multivariate linear regression between OCTA parameters and clinical data among all 115 CKD patients was presented in Table 5. β2-M was associated with thinner GC-IPLt (β: –0.294; 95% CI: –0.469∼–0.118), where PTH was associated with higher GCC-FLV (β: 0.004; 95% CI: 0.002∼0.006) and GCC-GLV (β: 0.007; 95% CI: 0.004∼0.01) among CKD patients. UACR was also found associated with higher GCC-FLV (β: 0.003; 95% CI: 0.001∼0.004) and GCC-GLV (β: 0.003; 95% CI: 0.001∼0.006). Besides, the results showed that the eGFR was positively associated with VD in SVP-WholeImage (β: 0.029; 95% CI: 0.017∼0.041), SVP-ParaFovea (β: 0.021; 95% CI: 0.002∼0.040), SVP-PeriFovea (β: 0.029; 95% CI: 0.016∼0.041), DVP-WholeImage (β: 0.033; 95% CI: 0.007∼0.058), DVP-ParaFovea (β: 0.044; 95% CI: 0.024∼0.065), and DVP-PeriFovea (β: 0.037; 95% CI: 0.008∼0.066).

**TABLE 5:** Stepwise multivariate linear regression between OCTA parameters and CKD-related parameters (115 CKD patients).

	eGFR		β2-M		UACR		PTH	
	β and 95% CI	*p*	β and 95% CI	*p*	β and 95% CI	*p*	β and 95% CI	*p*
SVP-WholeImage	0.029 (0.017∼0.041)	**<0.001**	–	–	–	–	–	–
SVP-ParaFovea	0.021 (0.002∼0.040)	**0.032**	–	–	–	–	–	–
SVP-PeriFovea	0.029 (0.016∼0.041)	**<0.001**	–	–	–	–	–	–
DVP-WholeImage	0.033 (0.007∼0.058)	**0.014**	–	–	–	–	–	–
DVP-ParaFovea	0.044 (0.024∼0.065)	**<0.001**	–	–	–	–	–	–
DVP-PeriFovea	0.037 (0.008∼0.066)	**0.013**	–	–	–	–	–	–
GC-IPLt	–	–	–0.294 (–0.469∼–0.118)	**0.001**	–	–	–	–
GCC-FLV	–	–	–	–	0.003 (0.001∼0.004)	**0.006**	0.004 (0.002∼0.006)	**0.001**
GCC-GLV	–	–	–	–	0.003 (0.001∼0.006)	**0.012**	0.007 (0.004∼0.010)	**<0.001**

*OCTA, optical coherence tomography angiography; CKD, chronic kidney disease; eGFR, estimated glomerular filtration rate; β2-M, β2-microglobulin; UACR, urine albumin to creatinine ratio; PTH, parathyroid hormone; CI, confidence interval; SVP, superficial vascular plexus; DVP, deep vascular plexus; GC-IPLt, ganglion cell-inner plexiform layer thickness; GCC, ganglion cell complex; FLV, focal loss volume; GLV, global loss volume.*

*Sex, age, BMI, history of hypertension, history of smoking, history of cardiovascular disease, eGFR, PTH, UACR, HGB, CKD duration, Hb1Ac, β2-M, and LDL were independent variables entered into the model.*

*The bold values indicated statistically significant (p value < 0.05).*

### Correlation Between Best-Corrected Visual Acuity and Optical Coherence Tomography Angiography Parameters

The results of correlation analyses of BCVA and OCTA parameters among all subjects were shown in Table 6. BCVA (LogMAR) was negatively correlated with VD in SVP and DVP (*r* = –0.399 ∼ –0.514, all *p* < 0.05), while positively with GCC-GLV (*r* = 0.201, *p* = 0.014).

**TABLE 6:** Correlations of retinal neurovascular parameters and BCVA (LogMAR).

	BCVA (LogMAR)			BCVA (LogMAR)	
Microvascular parameters	*r*	*P*	Neural parameters	*r*	*p*
SVP-WholeImage	–0.406	**<0.001**	GC-IPLt	–0.07	0.395
SVP-ParaFovea	–0.422	**<0.001**	GCC-FLV	0.114	0.166
SVP-PeriFovea	–0.399	**<0.001**	GCC-GLV	0.201	**0.014**
DVP-WholeImage	–0.488	**<0.001**	RNFLt-Average	–0.14	0.089
DVP-ParaFovea	–0.514	**<0.001**	RNFLt-Superior	–0.117	0.155
DVP-PeriFovea	–0.418	**<0.001**	RNFLt-Inferior	–0.072	0.384
RPC-WholeImage	–0.118	0.151			
RPC-WholeImage-Capillary	–0.008	0.919			

*BCVA, best-corrected visual acuity; SVP, superficial vascular plexus; DVP, deep vascular plexus; RPC, retinal peripapillary capillary; RNFLt, retinal nerve fiber layer thickness; GC-IPLt, ganglion cell-inner plexiform layer thickness; GCC, ganglion cell complex; GLV, global loss volume; FLV, focal loss volume.*

*The bold values indicated statistically significant (p value < 0.05).*

## Discussion

Two crucial findings were observed in this cross-sectional study. Firstly, our study revealed that retinal ganglion cell complex impairment occurs in the early stages (CKD stages 1–2) without detectable retinal microvascular rarefaction and RNFL damage in non-diabetic and non-dialytic CKD patients. Secondly, among CKD patients, the accumulation of uremic toxins of β2-M and PTH, and higher UACR was independently associated with retinal ganglion cell complex impairment, while reduced eGFR was associated with deceased retinal microvascular perfusion.

The major finding of this study is that increased GCC-FLV and GCC-GLV, and thinner GC-IPL could be detected in earlier CKD stages (stages 1–2), while no significant difference was found in all retinal microvascular parameters and RNFLt in the CKD 1–2 group compared to the control group, suggesting that retinal neuronal impairment has already existed in early CKD stages even in the absence of histologically assessed microvascular rarefaction and retinal axon damage. In fact, neurological complications, such as cognitive deterioration, cerebrovascular stroke, and cranial neuropathy, are common in CKD patients (Hamed, 2019). Recent population-based studies have documented an association between early stages of CKD and impaired cognition (Elias et al., 2009; Tsai et al., 2010). Given that the GC-IPL layer is where the retinal ganglion cell bodies are located, we postulated that it could serve as a window to observe CKD-related neuronal cell body loss in the CNS, particularly in the early stages of CKD. A recent population-based study consisting of 4464 non-glaucoma, multi-ethnic Asian participants showed that the presence of CKD is significantly associated with thinner GC-IPL (Tham et al., 2020). Furthermore, a prior study (Wu et al., 2020) also confirmed that macular thinning (GC-IPL and RNFLt) is a characteristic of retinal neural impairment in patients with CKD and such impairment is strongly associated with decreased eGFR. However, the definition of CKD applied in the aforementioned studies was based on an eGFR of <60 ml/min/1.73 m^2^ (CKD stages 3–5) and failed to include earlier stages CKD patients with an eGFR of > 60 ml/min/1.73 m^2^ (namely, CKD stages 1–2) using parameters such as proteinuria or UACR to detect early renal damage and declining renal function. Our study evaluated retinal neural parameters in non-diabetic CKD patients of stages 1–2 (versus health subjects) and revealed that retinal neuronal impairment, which might even precede microvascular rarefaction and retinal axon damage, could be detected in earlier CKD stages than those reported in prior studies (Jung et al., 2020; Wu et al., 2020).

On the other hand, significantly decreased RNFLt was observed in CKD patients according to previous studies (Demir et al., 2009; Jung et al., 2020; Wu et al., 2020). However, our study failed to detect significantly decreased RNFLt in either the CKD 1–2 group or the CKD 3–5 group compared to the control group. Two possible reasons may suffice to explain this conflicting result: firstly, significant reduced RNFLt were detected in end-stage renal disease patients which have been treated with dialysis in previous studies (Demir et al., 2009; Jung et al., 2020). But in our study, patients undergoing dialysis were excluded because dialysis itself might exert a significant impact on the neurovascular system. Thus, our results suggested that RNFL thickness is not reduced in non-dialysis CKD patients. Second, exclusion of DM patients may also play an important role. A previous study (Wu et al., 2020) reported significantly reduced RNFLt in CKD patients in stages 3–5. However, diabetic patients accounted for 42% of the CKD group in their study. It is reported that RNFLt could be significantly reduced even in preclinical DR stages (Zeng et al., 2019), suggesting that RNFL is highly susceptible to hyperglycemic injury. Diabetic patients were excluded from our study to avoid its strong impact on retinal neurovascular structures. Taken together, it could be speculated that RNFL damage might not be present in non-diabetic and non-dialytic CKD patients. With the progression of CKD, the RNFLt might reduce independently or secondary to GCC loss and retinal capillary rarefaction.

Stepwise multivariate linear regression was used to figure out independent risk factors associated with neurovascular alterations in the CKD patients of our study. It was shown that higher β2-M was associated with thinner GG-IPLt, and excessive PTH was associated with increased GCC-FLV and GCC-GLV, indicating the underlying pathophysiology of retinal neurodegeneration in CKD patients was related to the neurotoxic effects exerted by uremic toxins. In fact, as CKD progresses, there is the accumulation of organic toxic products which is considered highly associated with the incidence and progression of neurological complications (Hamed, 2019). Our results showed that GC-IPLt was independently associated with β2-M, after adjusting other confounding factors. The plasmatic level of β2-M increases as the kidney function gets impaired. The present study found that neuronal cell impairment in the retina was associated with the β2-M, which is supported by a previous study confirming the cytotoxic activity of β2-M on neuronal cell lines (Giorgetti et al., 2009). Excessive PTH can increase the influx of calcium into the brain which interferes with neurotransmission in the CNS and induces neurotoxicity (Cogan et al., 1978); therefore, it is considered to be neurotoxic and associated with cognitive dysfunction in CKD patients (Craver et al., 2007). Therefore, it is possible that GCC loss could be used as a biomarker to indicate or even predict CNS neuronal loss in CKD patients.

Another interesting finding is that higher UACR was independently associated with increased GCC-FLV and GCC-GLV. Previous studies have documented that albuminuria was independently associated with POAG (Kim et al., 2016), an ocular disease characterized by loss of the retinal ganglion cells in the retina (Shin et al., 2018), suggesting that albuminuria might be associated with retinal ganglion cell loss. In this regard, albuminuria (as indicated by higher UACR) might be a potential biomarker suggestive of neuronal damage in the retina. However, no association was found between UACR and decreased VD in SVP and DVP regions in CKD patients. In fact, the associations between albuminuria and retinal VD are controversial. A prior study reported that higher UACR was inversely related to SCP VD in non-diabetic hypertensive CKD patients (Vadala et al., 2019), while another research (Zhuang et al., 2020) showed that increased UACR was not associated with the VD in both SVP and DVP regions. Future studies are required to confirm the association between albuminuria and retinal VD alterations.

In addition, we also found that retinal VD decrease was detected in more advanced CKD stages (stages 3–5) and was associated with declined eGFR. This is consistent with the fact that microangiopathy and cardiovascular complications of CKD are also more prevalent in these patients (Houben et al., 2017). Thus, the retinal VD could serve as a biomarker to monitor the condition of systemic microvasculature in CKD patients and alert the risks of cardiovascular complications (Farrah et al., 2020). In fact, as the eGFR declining, the combination of uremia exposure (Zoccali et al., 2017), renal anemia (Chen et al., 2019), and impaired endothelial dysfunction compromise the structure and function of the systemic microcirculation (Ooi et al., 2011), which is considered a crucial pathway in the development and progression of cardiovascular complications of CKD (Houben et al., 2017). Our study also suggests that non-invasive imaging of the retinal vessels may reflect these systemic microvascular alterations, providing valuable information for identifying patients at risk of developing CKD-related cardiovascular complications.

There are two major implications of our findings. Firstly, strict control of uremia and close monitoring of neuronal impairment should be started in CKD patients at early stages (eGFR of > 60 ml/min/1.73m^2^), and parameters of the retinal ganglion cell complex layer can be used as indicators of early neuronal damage in these patients. Secondly, once retinal neuronal damage (such as GCC loss) is detected, the eGFR should be closely monitored to reduce CKD-related microangiopathy and other adverse cardiovascular complications.

There are two main strengths of our study. Firstly, by including patients with stages 1–2 CKD without diabetes and dialysis history, we were able to detect retinal neuronal impairment in early stages of CKD. Secondly, we also determine systemic risk factors associated with retinal neuronal damage and retinal capillary rarefaction in CKD patients. However, there are limitations in our study. Firstly, the cross-sectional design of our study prevented determining temporality and causal association between retinal neuronal impairment and microvascular hypoperfusion in CKD patients at different stages. The temporal sequence of the reported associations is needed to be validated in prospective cohort studies. Also, in patients with advanced stages of CKD, retinal microvasculature disease may be present even after kidney transplant (Ooi et al., 2015); it is important to explore more details about retinal neurovascular changes after renal replacement therapy. Secondly, the present study was also limited by its relatively limited sample size and further studies with larger sample sizes are required to confirm the findings in our future researches. Thirdly, some of the baseline variations, such as age and BMI, were not comparable among the three groups. Therefore, we adjusted them using binary logistic regression analysis to minimize the bias and made the results more reliable during the statistical analysis. Fourthly, we did not test the retinal function of CKD patients except visual acuity. In further investigations, we are going to evaluate retinal functions using multifocal electroretinogram (mfERG) in CKD patients to clarify the relationships between retinal neurovascular damages and retinal functions.

## Conclusion

Retinal neuronal impairment is present in early stages of CKD (stages 1–2) and it is associated with accumulation of uremic toxins and higher UACR, while retinal microvascular hypoperfusion, which is associated with worse eGFR, was only observed in relatively advanced stages of CKD (stages 3–5). The results highlight the importance of monitoring retinal neurovascular impairment in different stages of CKD.

## Data Availability Statement

The raw data supporting the conclusions of this article will be made available by the authors, upon request to HY, yuhonghua@gadph.org.cn.

## Ethics Statement

The studies involving human participants were reviewed and approved by the Research Ethics Committee of Guangdong Provincial People’s Hospital [registration number: GDREC2020069(R1)]. The patients/participants provided their written informed consent to participate in this study.

## Author Contributions

HY, XY, ZY, XZ, YH, and YC: conception and design. XZ, YL, BL, PZ, YX, CL, GW, HK, ZL, ZD, YR, and YF: analysis and interpretation of data. XZ, YH, YC, and ZL: drafting the manuscript and revising it. HY, XY, ZY, XZ, YH, and YC: providing intellectual content of critical importance to the work described. All authors approved the final version to be published.

## Conflict of Interest

The authors declare that the research was conducted in the absence of any commercial or financial relationships that could be construed as a potential conflict of interest.

## Publisher’s Note

All claims expressed in this article are solely those of the authors and do not necessarily represent those of their affiliated organizations, or those of the publisher, the editors and the reviewers. Any product that may be evaluated in this article, or claim that may be made by its manufacturer, is not guaranteed or endorsed by the publisher.
