# Supplementary material for: Retinal Neurovascular Impairment in Non-diabetic and Non-dialytic Chronic Kidney Disease Patients
Source: Front Neurosci. 2021 Nov 18;15:703898. doi: 10.3389/fnins.2021.703898 (PMC8639216; doi:10.3389/fnins.2021.703898)
Supplement: Supplementary file 1 [file Data_Sheet_1.pdf]

Supplementary materials-Figures

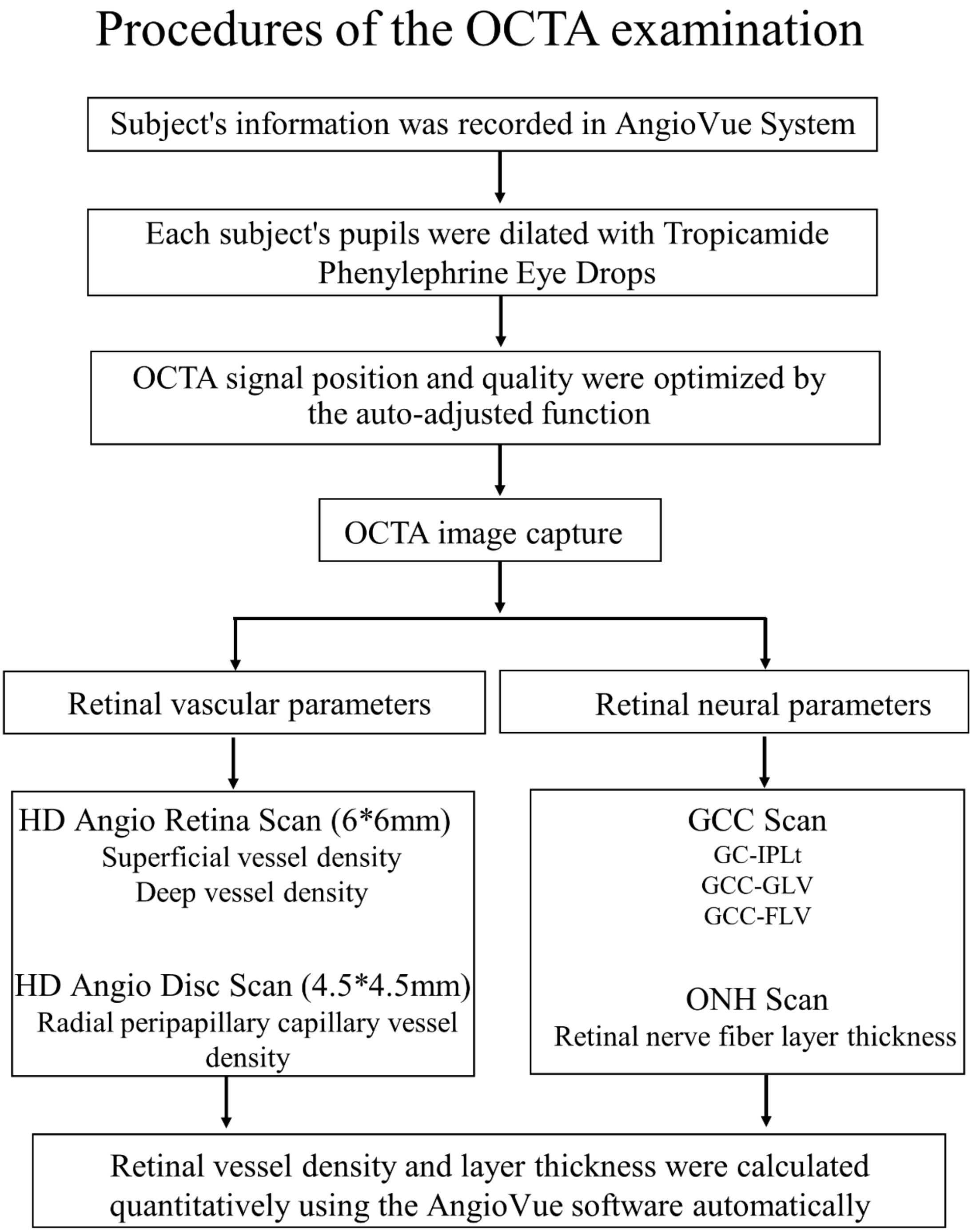

sFigure 1. Procedures of the OCTA examination.

Figure Legend: The flow chart showing the process of the OCTA examination.

Abbreviations: OCTA = optical coherence tomography angiography, HD = high definition, GCC = ganglion cell complex, GC-IPLt = ganglion cell-inner plexiform layer thickness; GLV = global loss volume, FLV = focal loss volume, ONH = optic nerve head

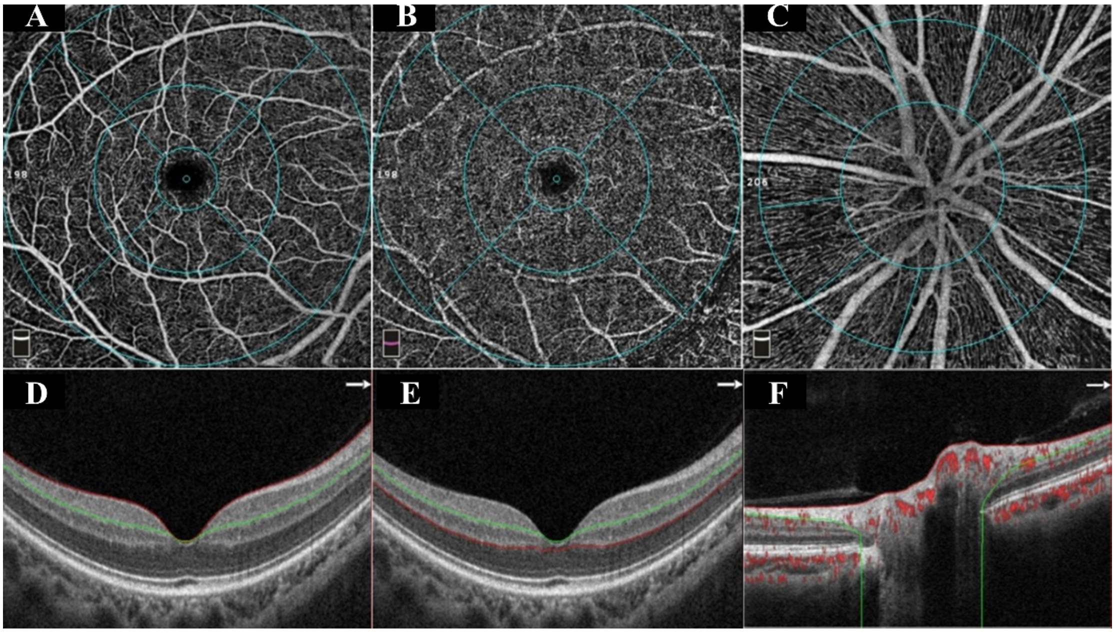

sFigure 2. OCTA en-face images show the representative picture of the three retinal vascular plexuses from a healthy right eye.

Figure Legend: (A) OCTA macula 6×6 scan showing the larger arteries and veins in the superficial vascular plexus; (B) OCTA macula 6×6 scan showing the lobular and tortuous capillary in the deep capillary plexus; (C) OCTA disc 4.5×4.5 scan showing the long and parallel capillaries of the radial peripapillary capillary network; (D) OCT B-scan showing the slab set to evaluate the superficial retinal plexus (from inner plexiform layer to 10 μm above inner plexiform layer); (E) OCT B-scan showing the slab set to evaluate the deep capillary plexus (from inner plexiform layer to 10 μm above inner plexiform layer); (F), OCT B-scan showing the slab set to evaluate the deep capillary plexus (from outer limit of retinal nerve fiber layer and internal limiting membrane).

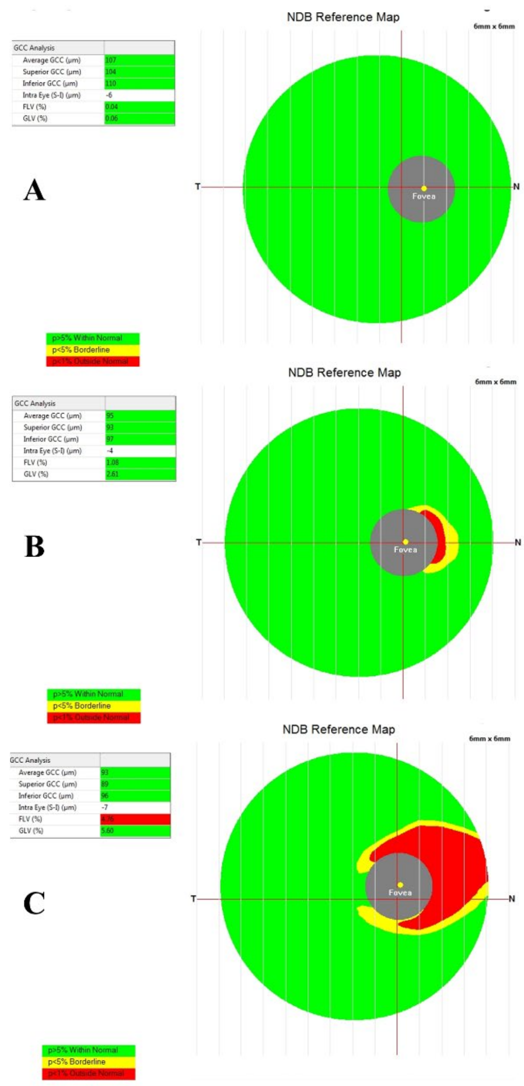

sFigure 3. A representative picture showing the changes in GC-IPLt, GCC-FLV and GCC-GLV in the CKD groups and control group.

Figure Legend: (A) showed the GC-IPLt of a 51-years old healthy man was within normal (Green area, the thickness > 5% of the normal subjects); (B) showed the GC-IPLt in a 49-years old male diagnosed with CKD stages 2. GC-IPLt in the partial areas on the nasal side were at the borderline (Yellow area, the thickness < 5% of the normal subjects) and in some outer areas on the nasal side were outside the normal limit (Red area, the thickness < 1% of the normal subjects); (C) showed the GCC-IPLt of a 50-years old male diagnosed with CKD stages 5. GC-IPLt in large outer areas on the nasal side were outside the normal limit (Red area, the thickness < 1% of the normal subjects). Abbreviations: GC-IPLt = ganglion cell-inner plexiform layer thickness, GCC = ganglion cell complex, GLV = global loss volume, FLV = focal loss volume, CKD = chronic kidney disease

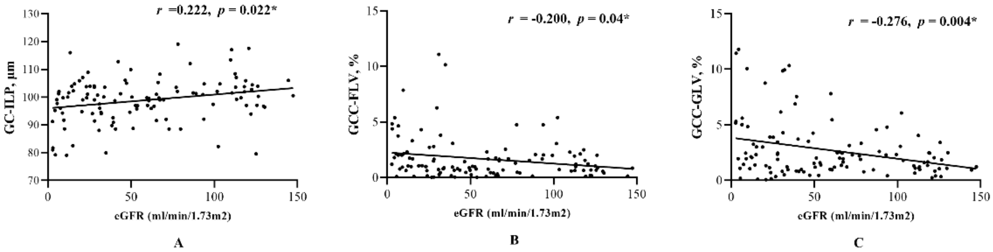

sFigure 4. Partial correlation of GCC variables and eGFR.

Figure Legend: (A), (B) and (C) were scatterplots showing correlations between GCC variables (GC-IPLt, GCC-FLV and GCC-GLV) and eGFR. Partial Correlation adjusted for age, sex, body mass index, history of smoking, history of cardiovascular disease, refraction error and intraocular pressure.

Abbreviations: GCC = ganglion cell complex, eGFR = estimated glomerular filtration rate, GC-IPLt = ganglion cell-inner plexiform layer thickness; GLV = global loss volume, FLV = focal loss volume
