# Supplementary material for: Retinal Neurovascular Impairment in Non-diabetic and Non-dialytic Chronic Kidney Disease Patients
Source: Front Neurosci. 2021 Nov 18;15:703898. doi: 10.3389/fnins.2021.703898 (PMC8639216; doi:10.3389/fnins.2021.703898)
Supplement: Supplementary file 2 [file Data_Sheet_2.pdf]

Supplementary materials - Tables

sTable 1. Etiology of CKD in 115 CKD patients

Etiology	Overall	CKD 1-2	CKD3-5	*p*
Primary glomerular diseases, n (%)	68 (59.1)	31 (62.0)	37 (56.9)	
Hypertensive nephropathy, n (%)	16 (13.9)	5 (10.0)	11 (16.9)	
Secondary glomerular diseases, n (%)	12 (10.4)	7 (14.0)	5 (7.7)	0.649
Renal tubulo-interstitial diseases, n (%)	10 (8.7)	4 (8.0)	6 (9.2)	
Other etiology, n (%)	9 (7.8)	3 (6.0)	6 (9.2)	

Abbreviations: CKD = chronic kidney disease
*p f*or the comparison between the CKD 1~2 group and the CKD 3~5 group using Pearson Chi-Aquare.

sTable 2. Comparison of the SSI among the three groups

	Control	CKD 1-2	CKD3-5	*p*
SSI - Macular region	8.54 ± 0.78	8.48 ± 0.84	8.2 ± 0.89	0.298
SSI - Disc region	75.7 ± 6.54	74.53 ± 7.05	73.59 ± 6.75	0.332
SSI - GCC region	76.13 ± 7.52	74.27 ± 7.23	74.16 ± 5.73	0.331
SSI - RNFL region	75.33 ± 7.76	75.73 ± 8.08	73.48 ± 6.71	0.294

Abbreviations: SSI = signal strength index, CKD = chronic kidney disease, GCC = ganglion cell complex, RNFL = retinal nerve fiber layer
